# Systems medicine and artificial intelligence in retinal disease

**DOI:** 10.1007/s00417-022-05868-3

**Published:** 2022-10-24

**Authors:** Oliver Zeitz, Sobha Sivaprasad, Antonia M. Joussen, Andrzej Grzybowski

**Affiliations:** 1 Department of Ophthalmology, Charité Universitätsmedizin Berlin, Augenklinik, Hindenburgdamm 30, 12203 Berlin, Germany; 2 Institute of Ophthalmology, NIHR Moorfields Biomedical Research Centre, Moorfields Eye Hospital, University College London, London, UK; 3 Department of Ophthalmology, University of Warmia and Mazury, Olsztyn, Poland; 4 Institute for Research in Ophthalmology, Foundation for Ophthalmology Development, Poznan, Poland

Ophthalmic research over the past decades was focused on clinical and preclinical analysis of ocular tissues. It neglected the fact that the eye is embedded into the biological system of the full human. System medicine is a novel approach, as it puts local pathologies into a broader context. It assumes that the course and even the development of localized disease process — be it an eye disease, heart disease, stroke or cancer or any other condition — is determined by the state of the entire body [9–11]. The genome is the underlying biological basis of each human. The totality of all messenger RNA represents the transcriptome. The proteome represents all genes that are ultimately expressed. The immunome represents the immune status of the subject and is an important modulator of processes involving immune responses and inflammation. The metabolome is the sum of metabolites and is highly dependent on external factors such as nutrition. The metabolome interacts with gene expression and the immune system. Further modulation comes from epigenetic factors as well as from the microbiome. Each of these systemic states has thousands of dimensions. E.g., the genome comprises 20,000 to 25,000 genes and the human proteome is estimated to include up to 400,000 different proteins. These numbers exemplify the complexity of systems medicine analysis.

Systems medicine carries significant potential for ophthalmology and retinal disease. Important conditions such as age-related macular degeneration (AMD), diabetic eye disease, retinal vascular occlusions, uveitis, central serous retinopathy (CSR), or glaucoma involve inflammation, neurodegeneration, or angiogenesis as key factors. These processes are not specific to the eye, yet they are universal processes of the entire body system. The way, e.g., how tissue inflammation manifests is determined by the complex state of the entire human body beginning with genetics and protein expression. Modulation by previous conditioning of the immune system, nutritive/metabolic factors, etc. Therefore, inflammation as the other processes is highly individual [1]. It is plausible to assume that these complex networks of factors also determine if and how an eye disease develops and how the course of the disease is. Systems medicine network analysis offers a new perspective on diseases of the eye. The concept of systems medicine can be expanded into further aspects of healthcare by leveraging its predictive, preventive, personalized and participatory character (P4 medicine) [3].

This new perspective may result in novel stratification schemes for ophthalmic disease. It may turn out that clinically similar disease states differ on a molecular level and require different treatments. Such development has taken place in the field of genetically caused retinal dystrophies. It is accepted and known today that very different genetic mutations may lead to similar clinical appearance or, vice-versa, that similar genetic mutations may lead to very different clinical appearance. In times of gene therapy, the molecular diagnosis is more important than the clinical diagnosis. Compared to retinal dystrophies, the complexity of AMD or CSR is much greater as these diseases are presumably caused by multifactorial combination of genetic, metabolic, and environmental factors [6]. Classic statistic methods are insufficient to decode these molecular networks. Here, machine learning and artificial intelligence come into play [7]. Unbiased methods for dimensionality reduction and other machine learning techniques may help to identify molecular fingerprints of subjects displaying certain disease features.

Machine learning (ML) and artificial intelligence (AI) are also used to exploit imaging data beyond what is visible for the eye of an ophthalmologist. AI-based technologies employing deep-learning (DL) approaches have proven effective in supporting decisions in many medical specialties, including radiology, cardiology, oncology, dermatology, and others. The number of studies using AI in the field of ophthalmology also has increased dramatically [4]. Although AI/ML algorithms can analyze different sort of data, its advantages were shown recently in detecting diseases based on image analysis, including chest radiographs, skin photos, and fundus photos and optical coherence tomography scans. They are already developed autonomous AI/ML medical devices registered in the USA and EU to detect diabetic retinopathy [5]. Interestingly, fundus photos have also been used to predict the risk of stroke, cardiovascular and kidney disorders. It was also shown that fundus photo can be a source of information previously unavailable to ophthalmologists, including age and gender, blood pressure, smoking, and body mass index [8]. The recent study reported that AI/ML algorithm trained on fundus pictures can predict risk of myocardial infarction [2].

In summary, systems medicine requires advanced statistics that are also used in the automated analysis of retinal imaging. Combining molecular and imaging data together has the potential to re-define eye disease. It may help to identify new targets for pharmacotherapy and may lead to a personalization of therapy based on molecular diagnoses.
